# Progress of Brain, Mind and Neurosciences in Universiti Sains Malaysia both Nationally and Internationally, and the Establishment of an Integrated Cerebrovascular Services and Clinic

**DOI:** 10.21315/mjms2023.30.6.18

**Published:** 2023-12-19

**Authors:** Muhammad Ihfaz Ismail, Song Yee Ang, Diana Noma Fitzrol, Zaitun Zakaria

**Affiliations:** 1 Department of Neurosciences and Brain Behaviour Cluster (BBC), School of Medical Sciences, Universiti Sains Malaysia, Kelantan, Malaysia; 2 Hospital Universiti Sains Malaysia, Universiti Sains Malaysia, Kelantan, Malaysia

Dear Editor,

We read with interest the Editorial article by Lee Yeong Yeh et al. in ‘The Brain-Gut Clinic in Hospital Universiti Sains Malaysia: Pioneering new service to advance neuro-gastroenterology and motility in Malaysia’ (1) and another Editorial article titled ‘Preparing Malaysia for population ageing through the advanced memory and cognitive service in Hospital Universiti Sains Malaysia’ (2). We congratulate both corresponding authors and report the vast advances in the Department of Neurosciences and the Brain Behaviour Cluster in the year 2023.

The human resource development for the country has produced a steady supply of neurosurgeons, clinical psychologist, cognitive neuroscientists and fundamental as well as applied neuroscientists (Figures 1–7).

The College of Surgeons of Malaysia was established in November 1972. It began with a founding membership of 23 fellows, led by Tan Sri Dato’ Dr. Abdul Majid Ismail as the first President. This milestone has played a pivotal role in shaping the history of surgery in Malaysia. The college is dedicated to upholding and advancing the highest standards of surgical practice within the country. Acknowledging the evolving responsibilities within the profession, in 1992, the fellows of the College, through a resolution, chose to merge with the Academy of Medicine of Malaysia. This strategic merger was officially ratified on 16 December 1995. Today, known as the College of Surgeons Academy of Medicine Malaysia (CSAMM), it serves as a representative body for diverse surgical specialties, actively promoting the advancement of the art and science across various surgical disciplines.

In 2023, CSAMM commemorates an important milestone by celebrating the Golden Jubilee Anniversary through its annual scientific congress. It’s with immense pride that CSAMM honours Professor Dato’ Dr. Jafri Malin Abdullah by bestowing upon him the 5th M. Balasegaram Trainer Award 2023 for his outstanding contributions to the field (Figure 8).

Continuing his trailblazing journey, Professor Dato’ Dr. Jafri Malin Abdullah adds yet another accolade to his notable career, securing the 4th Lifetime Achievement Award presented by the Neurosurgical Association of Malaysia. This esteemed recognition further solidifies his contributions to the field of neurosurgery. This prestigious recognition follows in the footsteps of luminaries such as Dato’ Dr. Johari Adan Serigar, Datuk Dr. Fadzli Cheah Abdullah and the inaugural recipient, Dato’ Dr. Selvapragasam (Figures 9a and 9b).

Transitioning from his notable achievements, Professor Dato’ Dr. Jafri Malin Abdullah was also seen as an active participant in the 78th session of the United Nations General Assembly (UNGA 78). During the noon session, which centred on Policies and Human Resources, the distinguished presence of Professor Dato’ Dr. Jafri Malin Abdullah was highlighted, standing alongside esteemed figures such as Argentinian Politician Neurologist Neuroscientist, Dr. Facundo Manes from the Instituto de Neurología Cognitiva (INECO) of Argentina and Dr. Walter Koroshetz, Director of the National Institute of Neurological Disorders and Stroke of the USA (Figure 10). The Department of Neurosciences and Brain Behaviour Cluster takes great pride in extending warm congratulations to Professor Dato’ Dr. Jafri Malin Abdullah for his ongoing contributions and continued success. He joined the Brain Health and the Brain Capital group recently by writing a policy paper that places Malaysia on the international map in this field (3).

Additionally, the Department of Neurosciences is thrilled to report the recent success of Professor Dr. Zamzuri Idris, who successfully conducted an awake surgery for epilepsy. This ground breaking achievement underscores our commitment to innovative approaches in neurosurgical procedures, further enhancing patient care and treatment outcomes (Figure 11).

Furthermore, the highlighting of Intraoperative Radiation Therapy with the INTRABEAM ZEISS system from Germany, employing a surgical approach followed by radiation has been conducted in Hospital Universiti Sains Malaysia. This innovative method was made possible by the presence of radiation physicists and oncologists. Notably rare, only a handful of leading global centres perform this procedure for tumours impacting the brain, breast, gastrointestinal tract, bladder and ENT. Gratitude to all involved contributors for their role in this advancement (Figure 12). Other advances in treatment includes brachial plexus and peripheral nerve surgery (Figure 13), superior temporal artery-middle cerebral artery (STA-MCA) bypass and carotid artery endarterectomy (Figure 14).

The integrated Neurosurgery-Neurology-Neurointervention services and clinic was established (Figures 15 and 16) on the return of Dr. Muhammad Ihfaz Ismail from his Cerebral vascular fellowship at Masaryk Hospital Usti nad Labem, Czech Republic under Professor Martin Sames and Ceske Budejovice Hospital, Czech Republic under Dr. Jiri Fiedler with the support of the World Federation of Neurological Societies (WFNS).

We extend our congratulations to the Hospital Sungai Buloh (HSB) Neurosurgery team on their groundbreaking achievement marking a significant milestone as the first implantation in the ASEAN region of the Directional DBS LEAD for Idiopathic Parkinson DBS surgery, conducted on 23 June 2023.

This remarkable progress is a testament to the exceptional teamwork between the Kementerian Kesihatan Malaysia HSB Neurosurgery, led by Mr. Saiful Azli Mat Nayan, Dr. Shahir and Neurologist at Hospital Kuala Lumpur (HKL), Ms. You Xin Li and their respective team members. Deepest gratitude is also extended to Department of Neurosciences Universiti Sains Malaysia (USM) team and Medtronic for their collaborative efforts on this historic achievement led by Professor Dato’ Dr. Abdul Rahman Izaini Ghani (Figure 17).

Congratulations are extended to the Integrated Neuroscience students for their successful thesis viva-voce defences and convocation day (Figures 18–21).

The Department of Neurosciences was honoured again nationally and internationally when Professor Dato’ Dr. Abdul Rahman Izaini Ghani was elected as the President of Neurosurgical Association of Malaysia (Figure 22) and Professor Dato’ Dr. Jafri Malin Abdullah was also being listed in the Top 2% in the World in Their Respective Fields (Brain, Mind and Neurosciences) by the Standford University (Figure 23).

The collobration of numerous Universiti Sains Malaysia lecturers, Ministry of Health Malaysia staffs as well as Universiti Pendidikan Sultan Idris (UPSI) lecturers have produced many new professionals for the country; even some have worked overseas in countries like the USA, Australia and Singapore.

We hope that with all these recent advances in clinical neurosciences by the Brain Behaviour Cluster and the Department of Neurosciences, School of Medical Sciences, Universiti Sains Malaysia, Health Campus in Kubang Kerian, Kota Bharu, Kelantan; we shall be a world leading clinical training institution to be proud of. In addition to that, an integrated handbook of Clinical Neurosurgery will be available by 2024, made specially for international surgical neurology residents.

## Figures and Tables

**Figure 1:**
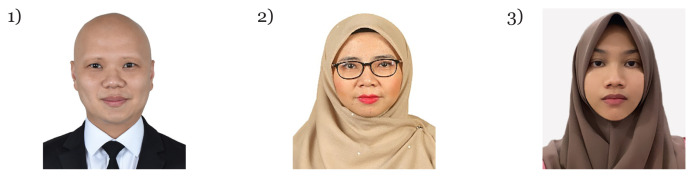
Master of Cognitive Neurosciences: 1) Kao Lin Ken, 2) Miza Hirdah Tahiruddin and 3) Sabrina Halimi

**Figure 2:**
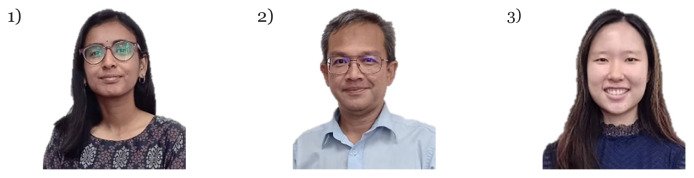
Master of Integrated Neuroscience: 1) Sutharshinnii a/p Murugan, 2) Ahmad Faris bin Razak and 3) Sherilyn Choo Ming Tze

**Figure 3:**
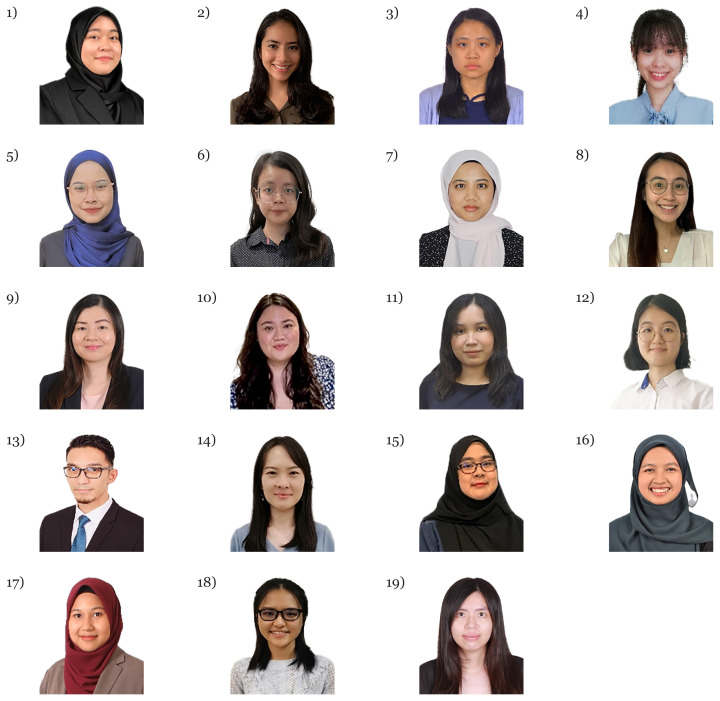
Master of Psychology (Clinical) (Cohort 5): 1) Aisyah Zulkipli, 2) Audrey Nathan Arul, 3) Cheong Jean Yi, 4) Chong Zi En, 5) Dayang Nursyazana, 6) Esther Goh Xin Yee, 7) Farah Umairah Sallehudin, 8) Ho Yen Ling, 9) Hue San Kuay, 10) Jasmine Elenore binti Amir Parrot, 11) Lidya Batrisyia Aderus, 12) Lim Zhi Ying, 13) Mohd Ashrawi bin Mohd Radzali, 14) Ng Lay Hong, 15) Nik Hafizah binti Zuraidi Afandi, 16) Nur Shafiqah binti Noor Ashani, 17) Siti Khadijah binti Asha’ari, 18) Susie Mun Hui and 19) Tan Saw Yen

**Figure 4:**
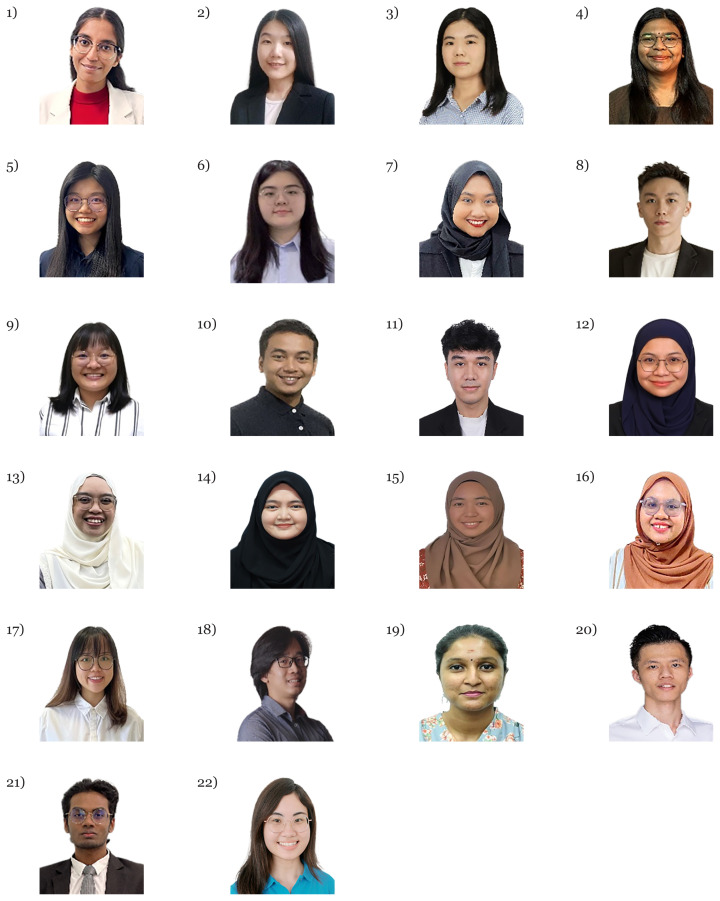
Master of Psychology (Clinical) Students Cohort 6: 1) Arginder Kaur, 2) Chon Yeng Yee, 3) Choong Hoay Chiing, 4) Deevaya Mugenthiran, 5) Haw Ying Huei, 6) Heo Xin Hui, 7) Iszati Afiqah Iskandar Zulkarnain, 8) Lim Zhen Chin, 9) Lo Siew Yau, 10) Muhamad Adam Mahamarowi, 11) Muhammad Hazzeq Shammim Azad, 12) Norsyafira Saad, 13) Nur Diana Zazana Mohd Esamuddin, 14) Nur Sobrina Aiman Ahmad Shukri, 15) Nurizzati Mohd Salleh, 16) Nursyahidatul Syahira Mazlan, 17) Oii Zeay Anne, 18) Reimy Tan, 19) Suwathi a/p Carmergam, 20) Tan Wei Jun, 21) Tinesh Subramaniam and 22) Vivian Lee

**Figure 5:**
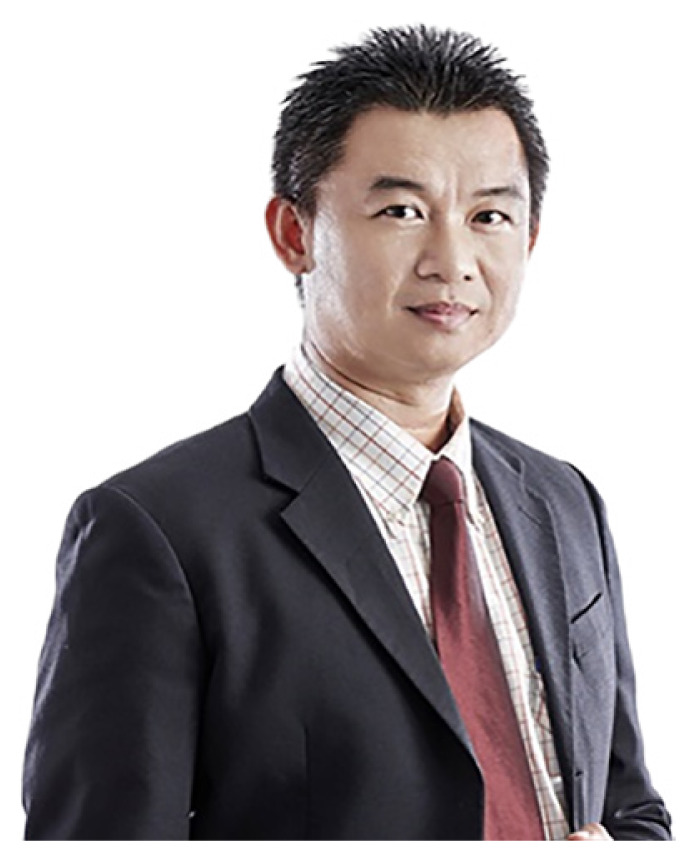
Doctor of Psychology (Clinical Neuropsychology) Student Cohort 6: Alex Ng Wei Siong

**Figure 6:**
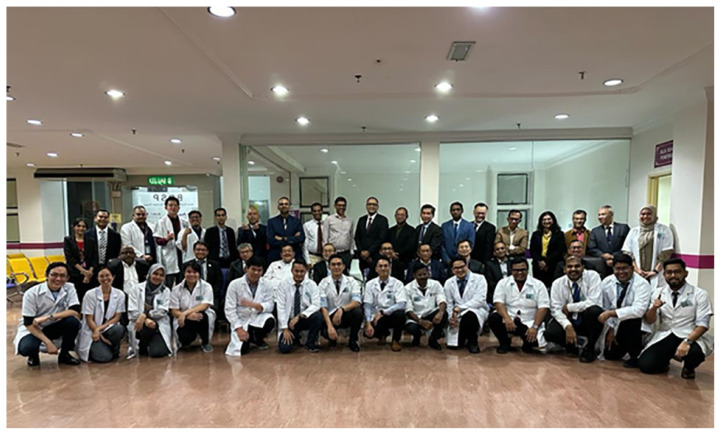
Our external examiner Professor Sri Maliawan from the Universiti Udayana of Bali, Indonesia with other examiners and candidates for First Conjoint Master Surgery (Neurosurgery) Part I, USM-UM 2023

**Figure 7:**
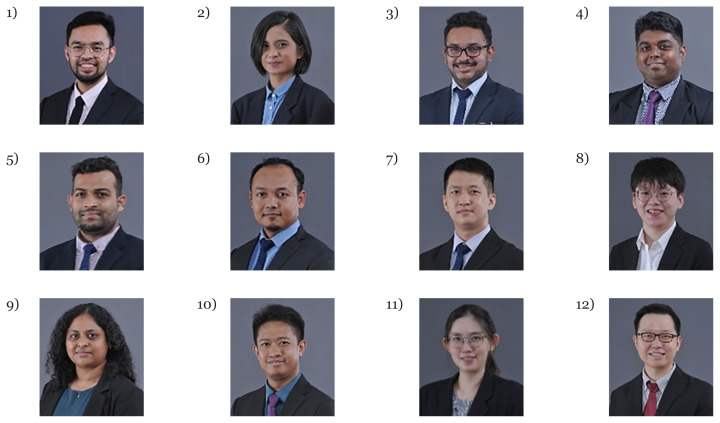
Master of Neurosurgery (intake May 2023): 1) Dr. Muhammad Safuan bin Sabri, 2) Dr. Atiqah Al-Aqilah Jamaluddin, 3) Dr. Thaanesh Manokaran, 4) Prehmanraj Mariyapan, 5) Muhammad Syamim bin Ali, 6) Dr. Muhamad Azuan bin Samsudin, 7) Dr. Heng Yu Wei, 8) Dr. Heng Pei Ting, 9) Dr. Darvena Pillay Sashideran, 10) Dr. Muhammad Zikri bin Yussof, 11) Dr. Chiun Pei Zhi and 12) Dr. Chin Wen Xin

**Figure 8:**
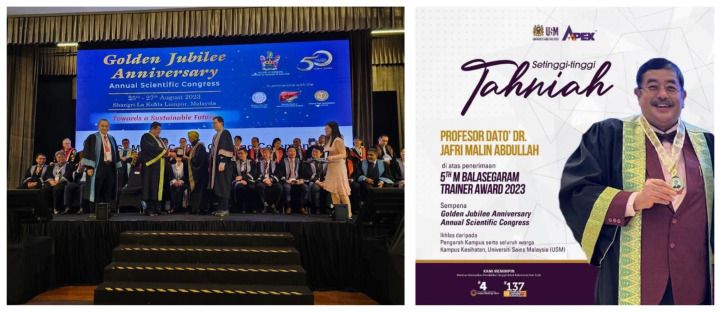
The prestigious M. Balasegaram award bestowed upon Professor Dato’ Dr. Jafri Malin Abdullah by the College of Surgeons of the Academy of Medicine Malaysia during their 50th anniversary

**Figure 9a:**
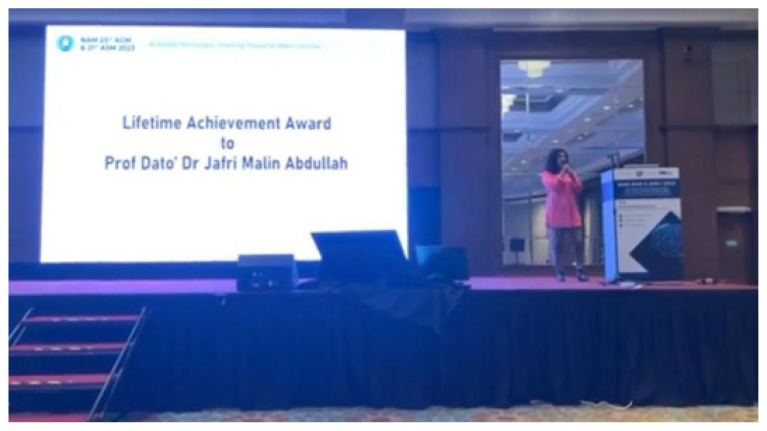
The 4th Lifetime Achievement Awards by the Neurosurgical Association of Malaysia after Dato’ Dr. Johari Adnan Serigar, Datuk Dr. Fadzli Cheah Abdullah and Dato’ Dr. Selvapragasam

**Figure 9b:**
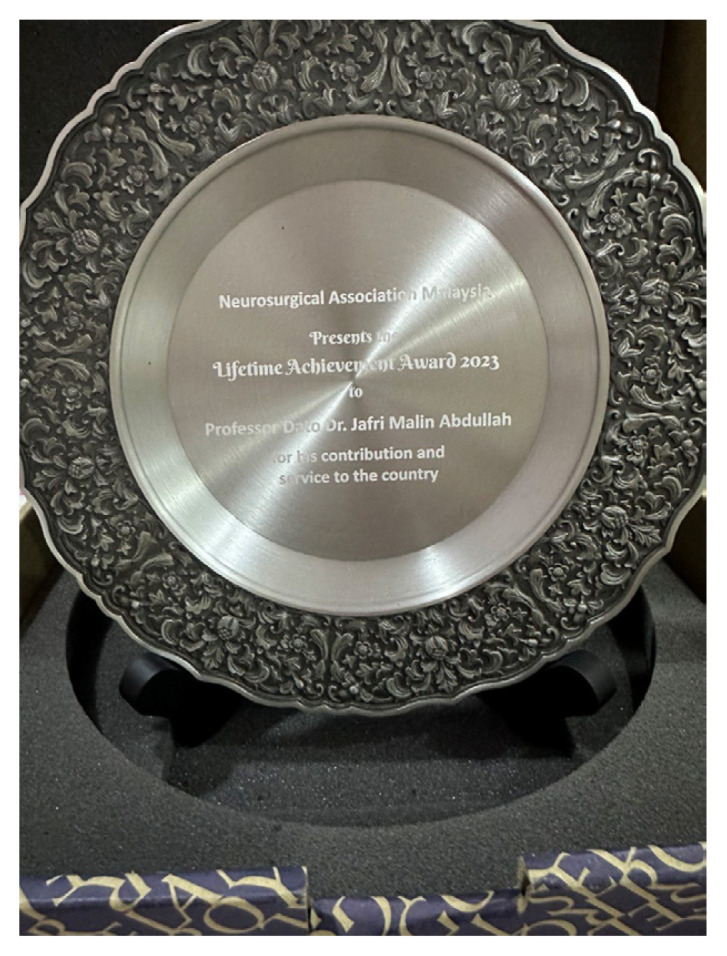
The Selangor Pewter plaque presented to Professor Dato’ Dr. Jafri Malin Abdullah

**Figure 10:**
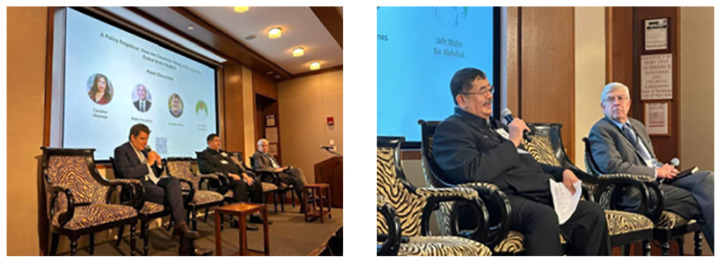
The noon session at UNGA 78 on Policies and Human Resources featured Professor Dato’ Dr. Jafri Malin Abdullah alongside Dr. Facundo Manes from the Instituto de Neurología Cognitiva of Argentina (on the left) and Dr. Walter Koroshetz, Director of the National Institute of Neurological Disorders and Stroke of the USA (on the right)

**Figure 11:**
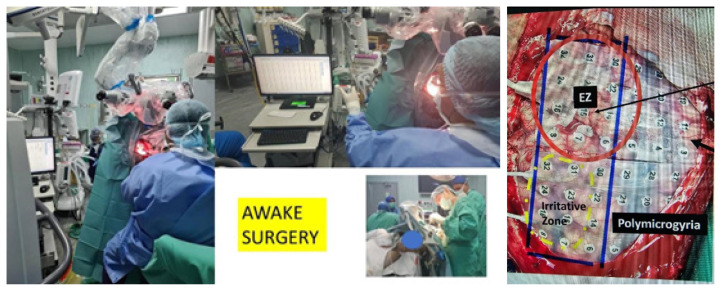
Professor Dr. Zamzuri Idris performing awake surgery for epilepsy surgery

**Figure 12:**
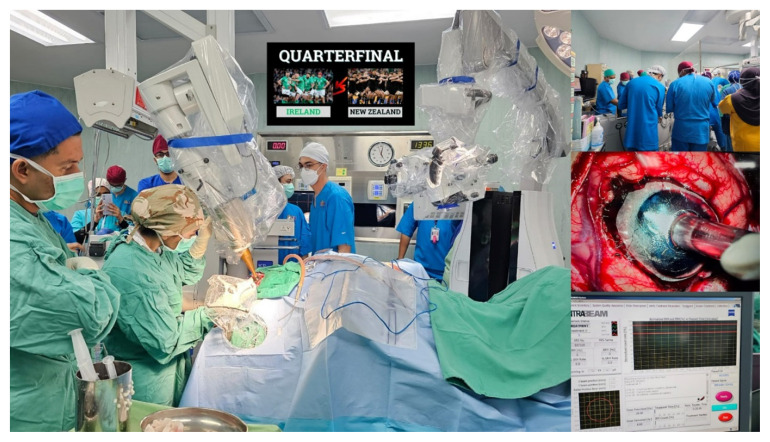
Intraoperative radiation therapy INTRABEAM ZEISS GERMANY surgery followed by radiation

**Figure 13:**
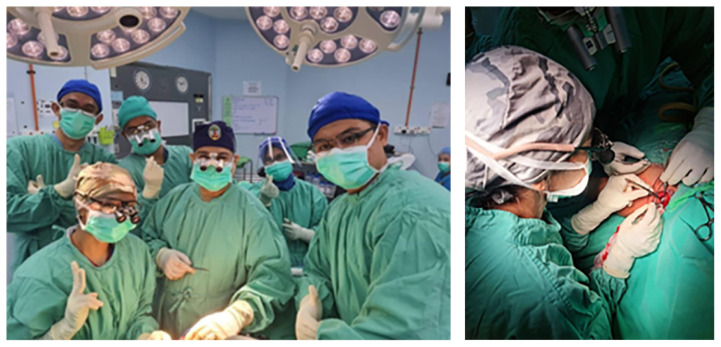
Collaboration in action: Dr. Diana Fitzrol, our neurosurgeon specialising in peripheral nerves, working alongside the Orthopedic team led by Associate Professor Dr. Abdul Nawfar Sadagatullah and Dr. Hanifah Jusoh during brachial plexus surgery

**Figure 14:**
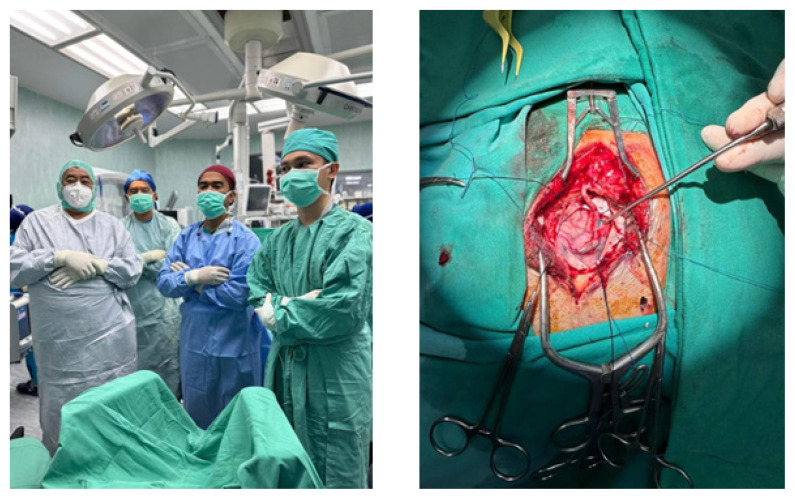
STA-MCA bypass surgery led by Dr. Muhammad Ihfaz Ismail, Professor Dato’ Dr. Jafri Malin Abdullah and Dr. Ang Song Yee (left); Post-STA-MCA bypass image presenting surgical outcomes (right)

**Figure 15:**
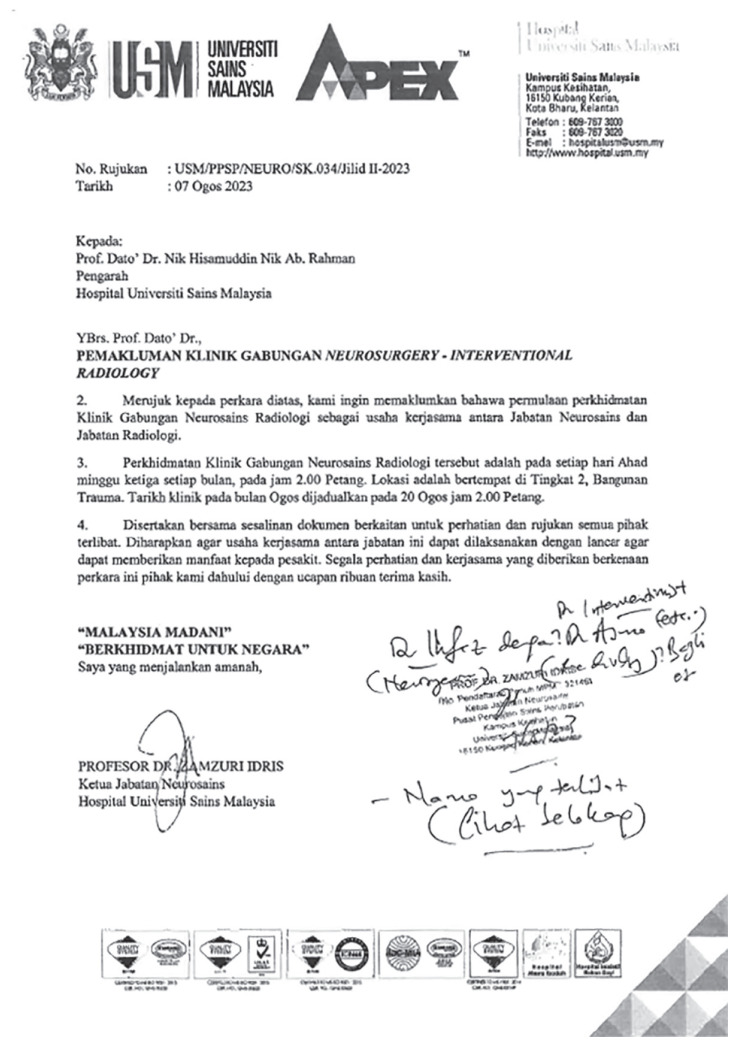
Approval letter for the Integrated Neurosurgery-Neurology-Neurointervention services and clinic

**Figure 16:**
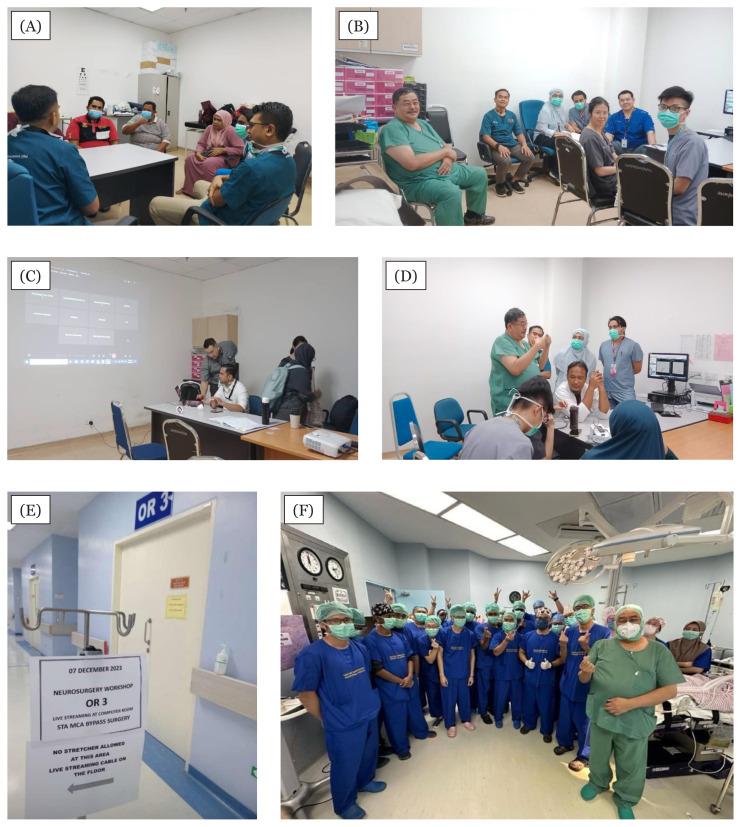
Among the activities at the Integrated Neurosurgery-Neurology-Neurointervention service and clinic. A) Dr. Muhammad Ihfaz Ismail and Dr. Bazli Md Yusoff of Interventional Radiology (IR) engaging in patient consultation during the combined Cerebrovascular Clinic, B) Professor Dato’ Dr. Jafri Malin Abdullah, Dr. Muhammad Ihfaz Ismail and neurosurgery residents collaborating during a clinic session, C) Multidisciplinary team discussing advanced management of Moyamoya disease with the Sabah Neurosurgery team via online telecast, D) Dr. Mohd Hafizudin Husin of IR and Professor Dato’ Dr. Jafri Malin Abdullah providing direct explanation to a patient during the clinic, E) We extended our service to do the first STA-MCA bypass of Moyamoya disease in Sabah on 7 December 2023 and F) Wonderful collaboration between Department of Neurosciences Hospital Universiti Sains Malaysia and Department of Neurosurgery Hospital Queen Elizabeth II, Kota Kinabalu Sabah; Professor Dato’ Dr. Jafri Malin Abdullah, Dr. Muhammad Ihfaz Ismail, Dr. Mohd Sofan bin Zenain (Head Department of Neurosurgery) Hospital Queen Elizabeth II, Dr. Hezry Abu Hassan, Dr. Ahmad Zulfadli Mohamaed Radzi, Dr. Harvinth Nagalingam Muniandy and Dr. Yeap Boon Tat (Neuroanaesthetist of Universiti Malaysia Sabah) between 6 December 2023 and 8 December 2023

**Figure 17:**
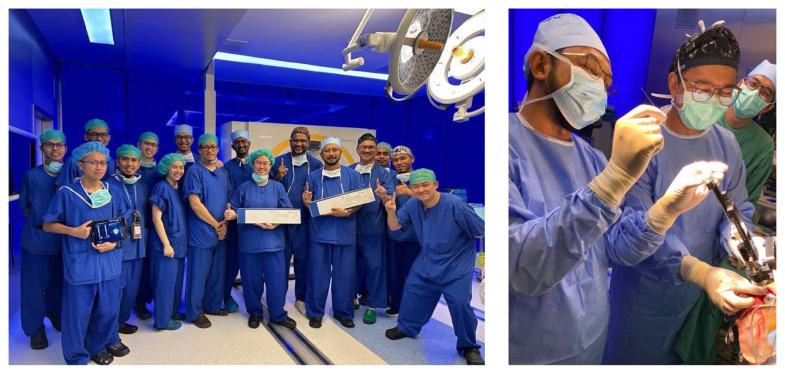
Historical event of the first implantation in ASEAN of Directional DBS LEAD for Idiopathic Parkinson DBS surgery, achieved through the collaborative efforts of KKM HSB Neurosurgery, HKL Neurologist, Department of Neurosciences USM team and Medtronic

**Figure 18:**
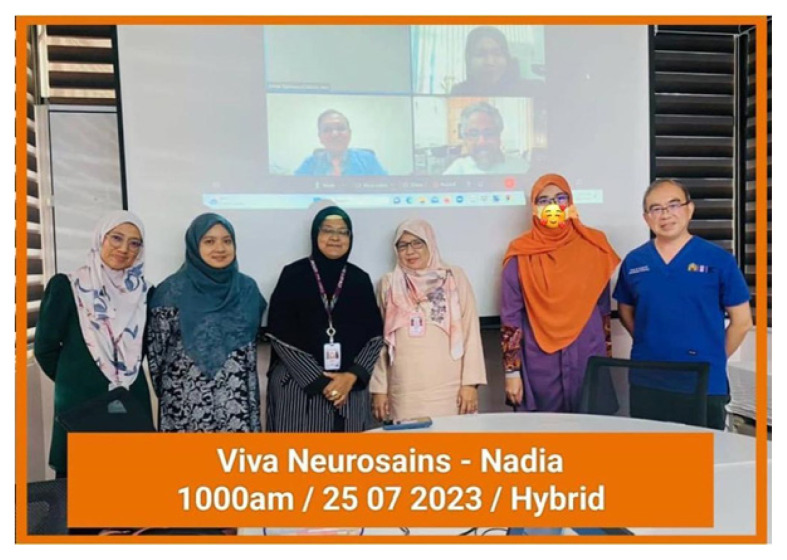
Thesis viva-voce examination on 25 July 2023

**Figure 19:**
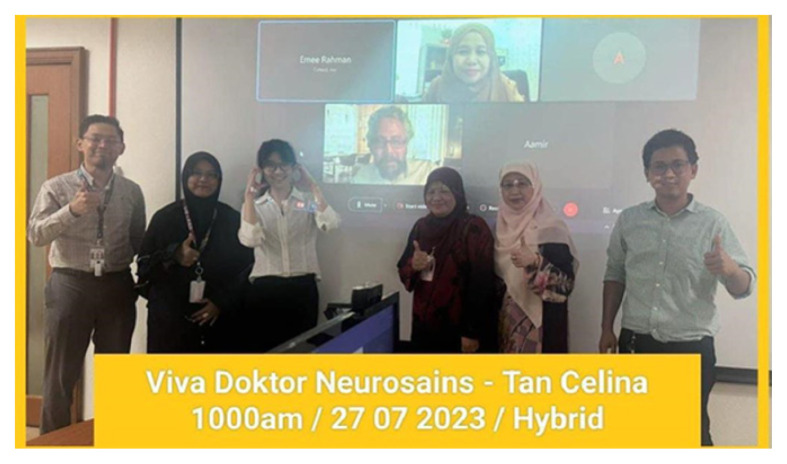
Thesis viva-voce examination on 27 July 2023

**Figure 20:**
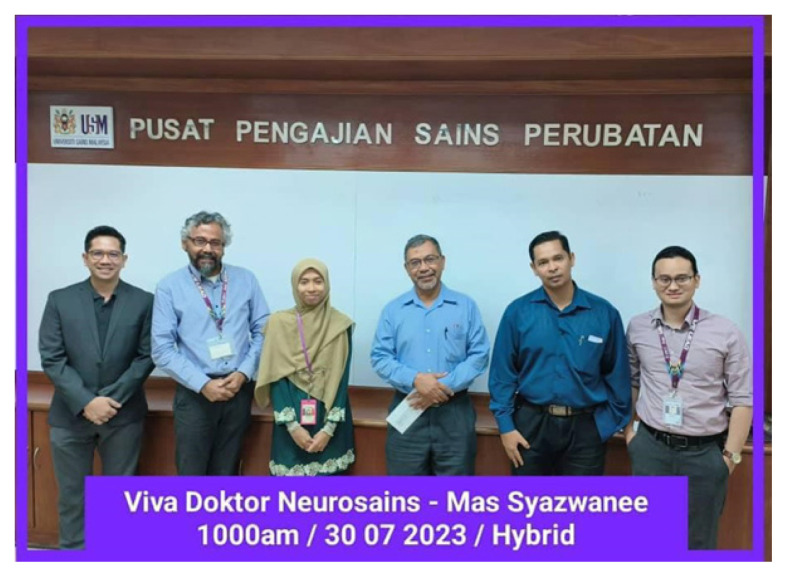
Thesis viva-voce examination on 30 July 2023

**Figure 21:**
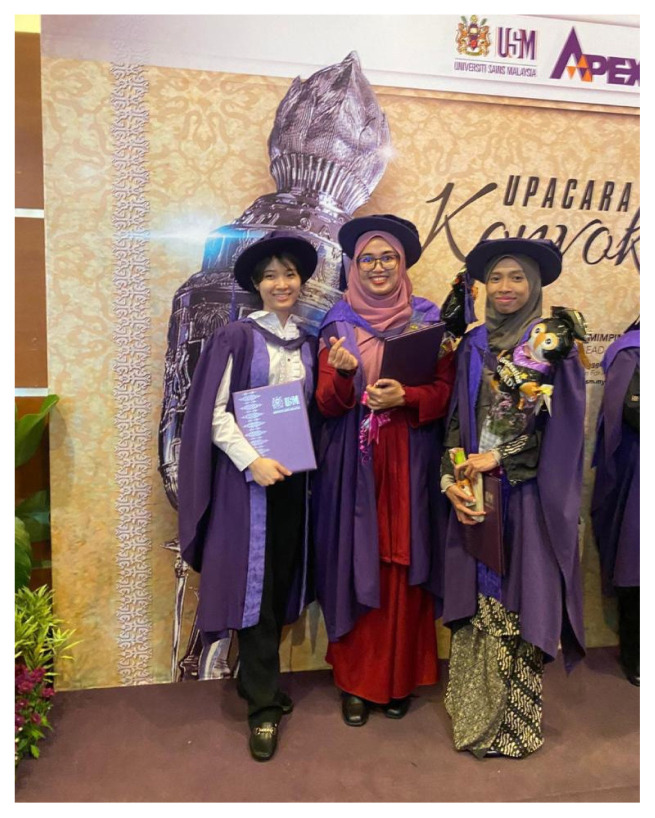
The 61st Universiti Sains Malaysia Convocation held recently on 22 November 2023

**Figure 22:**
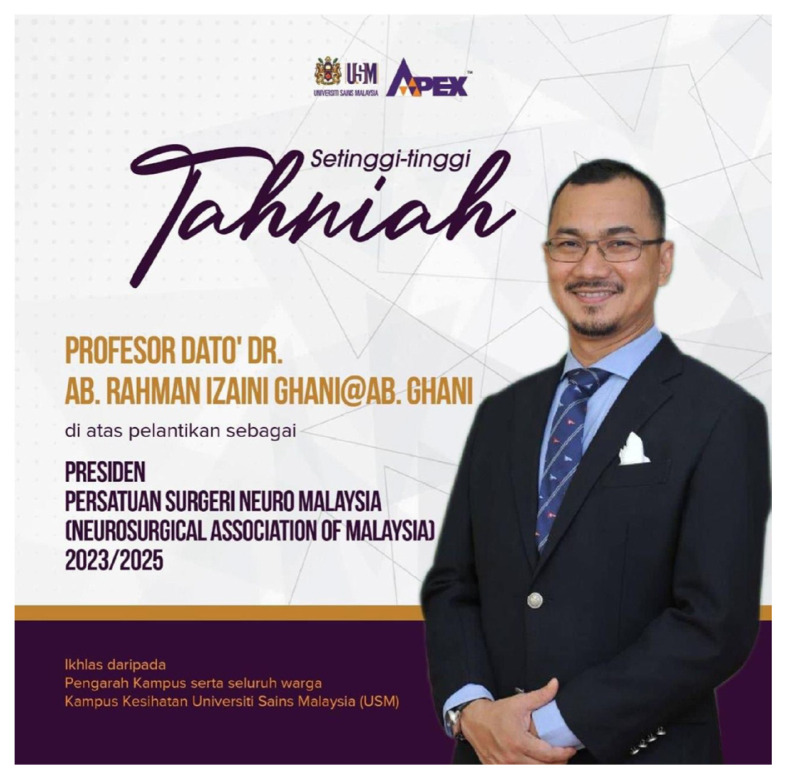
Congratulations to Professor Dato’ Dr. Abdul Rahman Izaini as the newly elected President of Neurosurgical Association of Malaysia (NAM)

**Figure 23:**
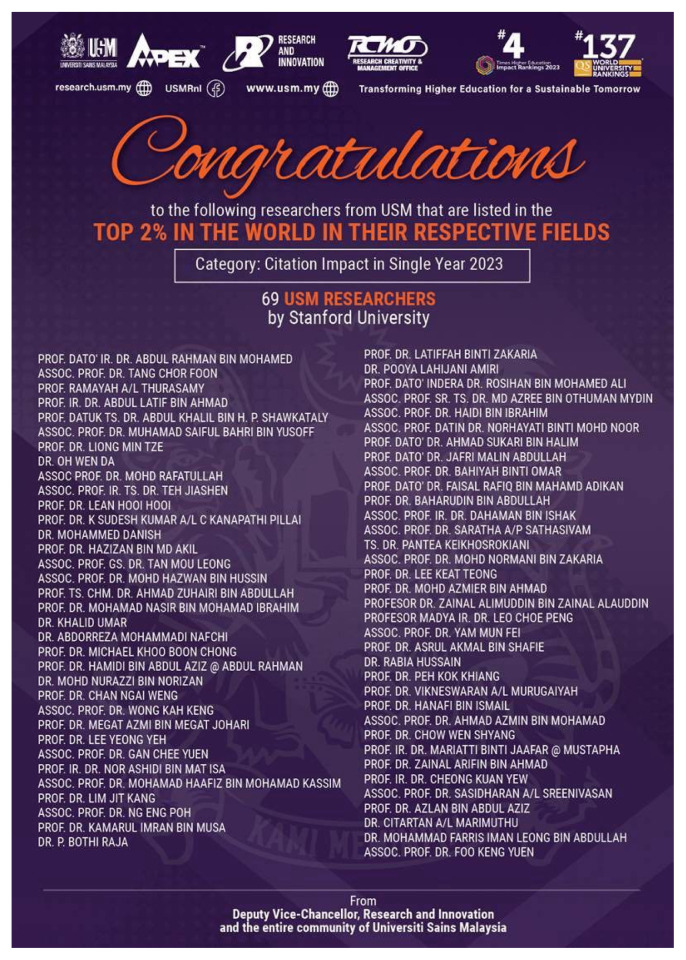
Professor Dato’ Dr. Jafri Malin Abdullah was listed in the Top 2% in the World in the field of Brain, Mind and Neurosciences by Standford University 2023
